# Items

**Published:** 1898-04

**Authors:** 


### Items.

---

Wyeth's Effervescent Salt of Phosphate of Sodium is described in our advertising pages. The quality of this preparation differs in many essential points from its class as generally presented, and is believed to be superior in many requisite qualities. It is a light, porous granular salt completely, and readily soluble with full free effervescence, copiously eliminating carbonic acid gas. It acts as a mild, cooling diuretic, aperient and stomachic stimulant. The bottle itself is a unique improvement, including measure-glass and time-dose record. The package at once suggests convenience, and we find on a practical, personal test that it has unusual merit. Physicians should ask to see, or apply to the manufacturers, Jno. Wyeth & Bro., Philadelphia, for specimens of this pleasant and useful laxative preparation.

---

Dr. William P. Spratling, superintendent of the Craig Colony, Sonyea, has proposed the formation of a National association for the study of epilepsy and the care and treatment of epileptics. It appears to us unnecessary to multiply medical societies or to divide the specialties. The care and treatment of epileptics come within the province of psycho-neurological associations, which afford an ample field for the study and consideration of epilepsy.

---

The M. J. Breitenbach Company, agents for the sale of Gude's Pepto-mangan, has been the victim of misrepresentation. Taking advantage of the fact that there is a sign-advertising company in New York bearing the name of Gude, that displays its name in bold letters in public places, evil-disposed persons have hinted that the two were one and the same concern. The Breitenbach company in a polite circular explains the whole matter to the profession. We invite attention to it at this time in the spirit of fairness and without solicitation of the company. Such practices are to be reprehended.

---

THE BEAUTIFUL ST. JOHN'S RIVER.—The Clyde Line, operating steamers between New York, Charleston and Jacksonville, Fla., in connection with Clyde's St. John's River Line, announce that on the 16th ult. they commenced running the well-known steamers between Jacksonville, Palatka, Sanford, Enterprise and intermediate points, leaving Jacksonville *daily* (except Saturdays). Returning these steamers leave the various landings daily (except Sundays) for Jacksonville, where connection is made with the Clyde Line steamers for the North. Heretofore the service on the St. John's River has been tri-weekly.

This interesting news will be hailed with delight by the numerous tourists and pleasure seekers who annually take in this delightful trip up the St. John's River. The beauties of the St. John's River have been heralded far and wide, and there are few Northerners who do not know of at least some of the attractions located on the banks of this river, the course of which is directed through the most tropical portion of Florida. In this section are located hundreds of boarding houses and hotels, where the choicest of Southern delicacies may be had.

The Clyde Line does the largest passenger business of any steamer line between New York and the South, and they have recently issued some very interesting literature, which they are sending out gratis to applicants. By addressing a communication to the Clyde Line, 5 Bowling Green, New York, our readers will in return be rewarded with some exceedingly attractive advertising matter regarding Southern resorts.

---

A UNIVERSITY day to be observed some time in June has been proposed by the University club of this city. It is believed that thus far no American city has made any effort to observe a special college day, and if the plans which were talked about at the University club are allowed to mature Buffalo will be the first city to originate the idea in America. The suggestion, which was made by the Rev. Lansing Van Schoonhoven, met with general approval. The Rev. Henry Elliott Mott and Mr. Henry H. Seymour, president of the club, spoke in favor of the plan. No definite arrangements were made, but if the scheme materialises all college men in the city will be invited to assist in the celebration. The suggestion is a good one and we hope it will be put into practical working.

---

THE New York Board of Trade and Transportation is making an investigation relating to the desirability of creating a national department of health and other methods of protecting public health as it affects interstate commerce. A special committee is conducting the inquiry on the following lines and invites suggestions, information and opinions thereon, such as they may be willing to make public, from experts and professional men having practical experience of sanitary affairs and from others having opinions on the subject.

1. Quarantine status and administration in foreign countries as furnishing precedents for the United States. (*a*) Border; (*b*) internal.

2. The present status of quarantine in the United States. (*a*) Border defense; (*b*) Interstate; (*c*) State and local.

3. The existing system of quarantine administration in the United States. (*a*) Cost; (*b*) Injury to and restrictions imposed on commerce and travel; (*c*) Security afforded.

4. Legislation needed for lessening injury to and restrictions on commerce and travel, and to afford greater security to the country. (*a*) Increase power of marine hospital service and how; or, (*b*) Create a national department of health; or, (*c*) Create a national department of commerce, with a bureau of health; or, (*d*) other suggestions.

5. The power of Congress under the Constitution to regulate matters affecting the health of the people—(*a*) National; (*b*) Interstate; (*c*) State and local.

Persons writing the committee are requested to affix to their names their professional letters, if any, and to address their communications to Mr. Silas M. Giddings, chairman, 203 Broadway, New York City.

---

THE Chutmuck Special is the name given to one of the handsomest railway trains ever operated over the Missouri Pacific line. This train will be used by excursionists to the meeting of the American Medical Association to be held at Denver next June. Attention is invited to the advertisement of the company on page xxxiii. For information relating to the Chutmuck Special, address H. C. Townsend, general passenger agent, St. Louis, Mo.

